# Pistil Starch Reserves at Anthesis Correlate with Final Flower Fate in Avocado (*Persea americana*)

**DOI:** 10.1371/journal.pone.0078467

**Published:** 2013-10-22

**Authors:** María Librada Alcaraz, José Ignacio Hormaza, Javier Rodrigo

**Affiliations:** 1 Instituto de Hortofruticultura Subtropical y Mediterránea “La Mayora”, Universidad de Málaga-Consejo Superior de Investigaciones Científicas (IHSM-UMA-CSIC), Estación Experimental “La Mayora”, Algarrobo-Costa, Málaga, Spain; 2 Unidad de Fruticultura, Centro de Investigación y Tecnología Agroalimentaria de Aragón (CITA), Zaragoza, Spain; University of Nottingham, United Kingdom

## Abstract

A common observation in different plant species is a massive abscission of flowers and fruitlets even after adequate pollination, but little is known as to the reason for this drop. Previous research has shown the importance of nutritive reserves accumulated in the flower on fertilization success and initial fruit development but direct evidence has been elusive. Avocado (*Persea americana*) is an extreme case of a species with a very low fruit to flower ratio. In this work, the implications of starch content in the avocado flower on the subsequent fruit set are explored. Firstly, starch content in individual ovaries was analysed from two populations of flowers with a different fruit set capacity showing that the flowers from the population that resulted in a higher percentage of fruit set contained significantly more starch. Secondly, in a different set of flowers, the style of each flower was excised one day after pollination, once the pollen tubes had reached the base of the style, and individually fixed for starch content analysis under the microscope once the fate of its corresponding ovary (that remained in the tree) was known. A high variability in starch content in the style was found among flowers, with some flowers having starch content up to 1,000 times higher than others, and the flowers that successfully developed into fruits presented significantly higher starch content in the style at anthesis than those that abscised. The relationship between starch content in the ovary and the capacity of set of the flower together with the correlation found between the starch content in the style and the fate of the ovary support the hypothesis that the carbohydrate reserves accumulated in the flower at anthesis are related to subsequent abscission or retention of the developing fruit.

## Introduction

 A common phenomenon in a wide range of flowering plants is the production of more flowers than fruits [1–5]. Different non-exclusive hypotheses, such as pollen limitation, pollinator attraction, bet hedging, selective abortion or pollen donation, have been proposed to explain this overproduction of flowers [1–3], and most of them could play a different role depending on the species studied and the conditions under which the experiments were performed. However, the influence that factors inherent to the flower could have in flower and fruitlet abscission has been little explored. 

Several studies suggest that seed and fruit abortion might be attributed to resource limitations [1,6,7]. In this sense, various reports have shown that the carbohydrate status of the plant is an important factor limiting yield [8]. Starch is the most important storage carbohydrate in plants, especially in woody perennial species [9] and changes in starch content in different plant structures play an important role as indicators of sink and source activities [10] and are closely related to changes in the physiological activity of a particular organ or tissue [11]. A low carbohydrate availability could, for example, influence yield by reducing the number of flowers and this has been shown by a positive correlation between starch content in the wood at bud break and the number of flowers developed [12–14]. Carbohydrate availability could also influence yield by adjusting fruit number to the metabolite supply of the tree through the premature abscission of flowers and developing fruits.

In woody plant species, the carbohydrate support needed for flower and fruit development originates either from reserves accumulated in the tree the year before or from photosynthesis performed in the inflorescences, flowers or leaves [15–17]. Moreover, the events that occur between pollination and early fruit set are highly dependent on the starch content present in the different structures of the pistil [17–22]. Different lines of evidence strongly suggest that starch content in the pistil is crucial for subsequent ovule fate [7,23–25].

Avocado (*Persea americana* Mill.), a member of the Lauraceae, a family included in the early-divergent Magnoliid clade in the angiosperms, is an extreme case of a species with a very low fruit to flower ratio, with less than 1% of the flowers produced able to set fruits due to a massive drop of flowers and developing fruitlets [26–29]. The avocado flower is bisexual having both functional male and female organs although separated in time through a synchronous protogynous dichogamous breeding system that prevents self-pollination promoting outcrossing. Each flower opens twice, the first functionally as a female flower with a white receptive stigma; the flower closes overnight and reopens the following day functionally as a male flower [30]. The species shows heterodichogamy since the different avocado cultivars can be classified in two groups (A or B) based upon their flowering behaviour [31]. In type A cultivars, such as ‘Hass’, the flowers open in the morning in the female stage, close at midday and reopen the afternoon of the following day at the male stage. In type B cultivars, such as ‘Fuerte’, the flowers open in the afternoon at the female stage, close in the evening and reopen the following morning at the male stage [32].

Although different factors such as temperature [33,34], outcrossing rate [35,36], water and nutrient availability [37] and alternate bearing [29] have been proposed as limiting factors in avocado fruit production, information is still elusive to explain why most flowers prematurely abscise. In avocado, as in other evergreen woody plant species, vegetative and reproductive growth occur simultaneously and, consequently, vegetative and reproductive organs compete for resources provided by either reserve mobilization or photosynthesis [38,39]. Previous works in avocado [40] and other species [39,41] have suggested that this competition may be important in determining flower abscission.

In avocado, starch is present in the ovary at anthesis [42], although a wide variability among flowers in starch content within the pistil has been recently reported [43]. This starch stored in the pistil at anthesis has been related to initial ovary growth [43]; however, the implications of these reserves in flower fate have not yet been analyzed. While indirect evidence in different species supports the statement that the carbohydrate reserves in the flower are related to subsequent abscission or retention of the developing fruit, no direct evidence has been yet reported. This can be due to the difficulty in relating the nutritive status of a flower to its future fate [22] or because the analyses of carbohydrate content of flowers are performed when the developing fruitlets had finally abscised or set, and carbohydrate reserves had already diminished compared to the initial stage at flowering [22,44]. As an alternative, in this study the implications of starch in the avocado flower on the subsequent fruit set are explored by measuring the starch content of the ovary and the style and relating it to the abscission or survival of the flower. As a first step, starch content in the ovary was evaluated in individual flowers from two different populations (early and late flowers) with a different capacity to set fruit within the same trees. Secondly, pollen tube growth and starch content in the style were sequentially examined in the days after anthesis. Finally, in a set of randomly selected flowers the style was excised above the ovary, after the living tip of the pollen tubes had passed through, and final fruit set was evaluated. This allowed us to determine the relationship of the starch content of each detached style to the fate of the ovary of the same flower that remained on the tree. 

## Materials and Methods

### Plant material

 The experiments were carried out in 25 years old ‘Hass’ avocado trees, the most important commercial cultivar worldwide, in the experimental orchards of IHSM-UMA-CSIC La Mayora (Málaga, Spain), which is a public institution for agricultural research and where no specific permissions are required for this kind of field studies. During the flowering period, which in ‘Hass’ takes place in April and early May [26], the average values of mean, maximum and minimum temperature were 16.1°C (range: 12.3°C -19.5°C), 20°C (15°C -24°C), and 12.2°C (8°C -15°C), respectively.

### Field experiments

#### Early and late flowering inflorescences

Under the environmental conditions of Southern Spain a second wave of inflorescence development often takes place about one month after the main flowering period. This second wave of inflorescences, however, usually does not produce fruits. Therefore, comparing the starch content in flowers from inflorescences of the two periods could provide relevant information for the understanding of the implications of starch content in the ovary at anthesis on final flower fate. 

To compare the nutritional status of those two different types of inflorescences, 30 early-developing inflorescences and 10 late-developing inflorescences from 3 and 5 trees respectively were labelled. Ten flowers at anthesis of each inflorescence were collected every day and fixed in FAA (70% ethanol: glacial acetic acid: formalin [18:1:1; v/v/v]) [45]. Ninety flowers from early-developing inflorescences and 30 flowers from late-developing inflorescences were randomly selected and their starch content was measured under the microscope (see below for methods). Differences in starch content between flowers from early and from late-developing inflorescences were evaluated using a Student’s t test at the 0.05 significance level. Statistical analyses were performed using SPSS 17.0 statistical software (SPSS Inc., Chicago, IL).

To estimate the percentage of fruit set, 60 early-developing and 10 late-developing inflorescences were labelled and left to undergo open pollination. Weekly counts of flowers and developing fruits were monitored from anthesis to harvest in both populations of inflorescences. No statistical analysis was applied as no fruits developed from late inflorescences (see Results). 

#### Timing of style removal

In order to optimize the period when the styles could be excised without affecting fertilization a preliminary experiment was performed to establish the time of pollen tube arrival at the base of the style. For this purpose, flowers of ‘Hass’ at anthesis obtained from 10 inflorescences were hand-pollinated by direct contact of the anthers of flowers of ‘Fuerte’. At least 20 pistils were collected 2, 4, 6, 8, 24, 30 and 48 h after pollination and fixed in FAA, to analyse pollen tube behaviour under the microscope (see below for methods).

The number of pollen tubes was counted in four different regions of the style, which were defined by dividing the length of the style into quarters: one next to the stigma (1/4); one at the base, next to the ovary (4/4); and two in between (2/4 and 3/4). After checking for normality and homocedasticity in data, repeated measures ANOVA, with “region of the style” as repeated measure and “time after pollination” as independent variable, was used to determine how long after hand pollination pollen tubes reached each region of the style. When the assumption of sphericity was not met (Mauchly’s sphericity test at *P* < 0.05), degrees of freedom were adjusted following the Greenhouse-Geisser (G-G) correction. Because significant between*within subject effects were found (see Results), univariate ANOVAs were subsequently performed to ascertain, by comparing the time needed for pollen tubes to reach each of the regions, when styles could be removed without interfering with fertilization. ANOVA’s were followed by Tukey’s post-hoc tests.

#### Mobilization of reserves in the style

In order to characterize the mobilization of reserves in the style, the starch content of the style was examined following anthesis. For this purpose, 100 pistils were hand pollinated at anthesis, and 10 pistils per day from anthesis to 9 days later were collected and fixed in FAA. The starch content in the style was then measured in 4-7 pistils randomly chosen out of the 10 collected per day. Data were analysed with one-way ANOVA, followed by Tukey-Kramer post-hoc test for unequal group sizes.

#### Starch content in the style and fruit set

In a different experiment, the nutritional status of individual flowers was analysed and related to the subsequent set or abscission of the corresponding ovary that remained in the tree. For this purpose, 25 inflorescences were selected in 3 trees of ‘Hass’ (flowering type A), in which 597 flowers were individually labelled and hand pollinated with pollen of ‘Fuerte’ (flowering type B). The style of each flower was excised above the ovary one day after pollination, once the living tip of the pollen tubes had passed through based on the results of the experiment where optimal timing for style removal was evaluated (see above). Each style was individually fixed in FAA for further analysis. The timing of flower and fruit abscission was recorded weekly from the end of the flowering season until the fruits reached maturity. This allowed the analysis of the starch content of styles in each individual flower while knowing the fate of the corresponding ovary that remained on the tree. A Student’s t-test at the 0.05 significance level was used to compare the starch content of styles from flowers that dropped with that from flowers that set fruits.

 In order to determine whether style removal could have influenced flower abscission or retention, fruit set was also monitored in another set of 645 hand-pollinated flowers from 20 inflorescences from the same trees under the same conditions as treated flowers but without excision of the styles. A Chi-square test for 2 x 2 contingency tables, with Yate´s correction for continuity, was used to compare the percentages of fruit set in flowers which styles had been excised and in flowers that had not. 

### Pollen tube growth in the style

Pollen tube growth was monitored on squashed preparations. Thus, the fixed pistils were washed three times with distilled water, for an hour at a time, and left overnight in 5% sodium sulphite. On the following day, the pistils were autoclaved at 1 Kg / cm^2^ for 10 min in 5% (w/v) sodium sulphite to soften the tissues and the pistils were then stained with 0.1% (v/v) aniline blue in 0.3 M K_3_PO_4_ for callose [46]. Preparations were observed under a Leica DM LB2 microscope with UV epifluorescence using a BP 515-560 exciter filter and an LP 590 barrier filter. 

### Evaluation of starch content

 The presence of starch was monitored in ovaries and/or styles of the flowers analysed. For this purpose, the plant material that had been fixed in FAA was dehydrated in a tertiary butyl alcohol series (70, 85, 95 and 100% v/v), embedded in paraffin wax and sectioned transversally at 10 μm in an AS325 retraction microtome (Shandon, UK). Prior to staining, the sections were rehydrated [three washes in Histoclear (CellPath, Hemel, UK), one in Histoclear:ethanol (1:1, v/v) and one in an ethanol series (100, 70 and 40%, v/v)]. The samples were then stained using the potassium iodide-iodine reaction (I_2_KI) for 5 min and observed under an Ortholux II microscope (Leitz, Wetzlar, Germany). The images of I_2_KI stained preparations were collected using a DC300 digital camera connected to the microscope and processed using a Quantiment Q550 Image Analysis (Leica Microsystems CMS GmbH, Wetzlar, Germany). The procedure is based on the measurement of the optical density of black and white images using a method previously described [47] with modifications [43]. 

In the experiment comparing early and late-developing inflorescences, measurements were taken in the cortical tissue of the ovary wall because starch content in this area reflects accurately the starch content in the pistil of avocado flowers [43]. Measurements of starch content were taken in two different sections in each ovary, i.e. in the largest and in the smallest section of the ovule. In each ovary section, four measurements of the optical density of the stained starch were taken made within a frame of 1,337 µm^2^. The average value of these eight values was taken as the estimate of the starch content of the ovary. The starch content was measured in 90 flowers collected from early-developing inflorescences and in 30 flowers from late-developing inflorescences.

**Figure 1:**
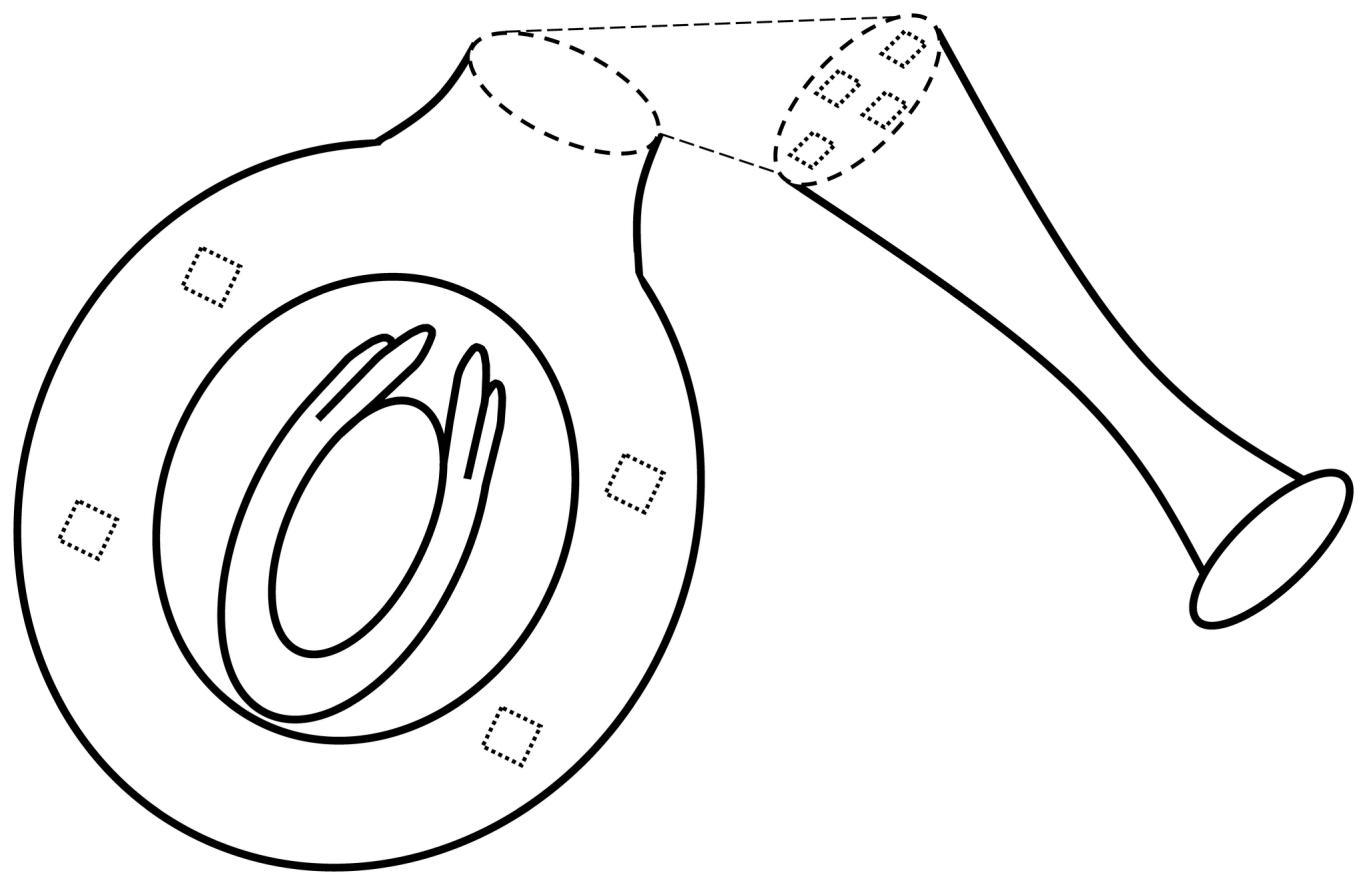
Diagram representing the pistil of an avocado flower after the dissection of the style. Boxes indicate the regions in which starch content was measured in the ovary and cortical tissue of the style with the image analysis system.

 Prior to performing the experiment to compare the nutritive status of the style in abscising and non-abscising flowers, and to determine whether the starch content of the style was correlated to the starch content in the different tissues of the ovary, 150 flowers at anthesis were randomly collected from three trees and their starch content was evaluated at different levels in the upper and lower sections of the ovary and in the style (Figure 1). In every section analysed, four measures of the optical density of the stained starch were taken in a frame of 1,337 µm^2^ and the average value of each structure was used as the estimate of its starch content. However, starch content estimates were highly variable among flowers (see Results). Therefore, to detect a putative relationship between the starch content in the cortical tissue of the style and that in different parts of the upper and lower sections of the ovary and the transmitting tissue of the style at anthesis, while correcting for differences among flowers, Pearson’s correlation coefficients at the 0.01 significance level were computed.

 In the experiment to compare the starch content of the style in abscising and non-abscising flowers, once the fate of each ovary remaining on the tree after style dissection was known in the 597 individually labelled flowers, the starch content of the style was analysed from 15 flowers that finally set fruits and from 40 randomly selected flowers that finally abscised. Student’s t-test at the 0.05 significance level was used to detect differences in starch content between the population of flowers that dropped and the population of flowers that set fruits.

## Results

### Avocado progamic phase

**Figure 2:**
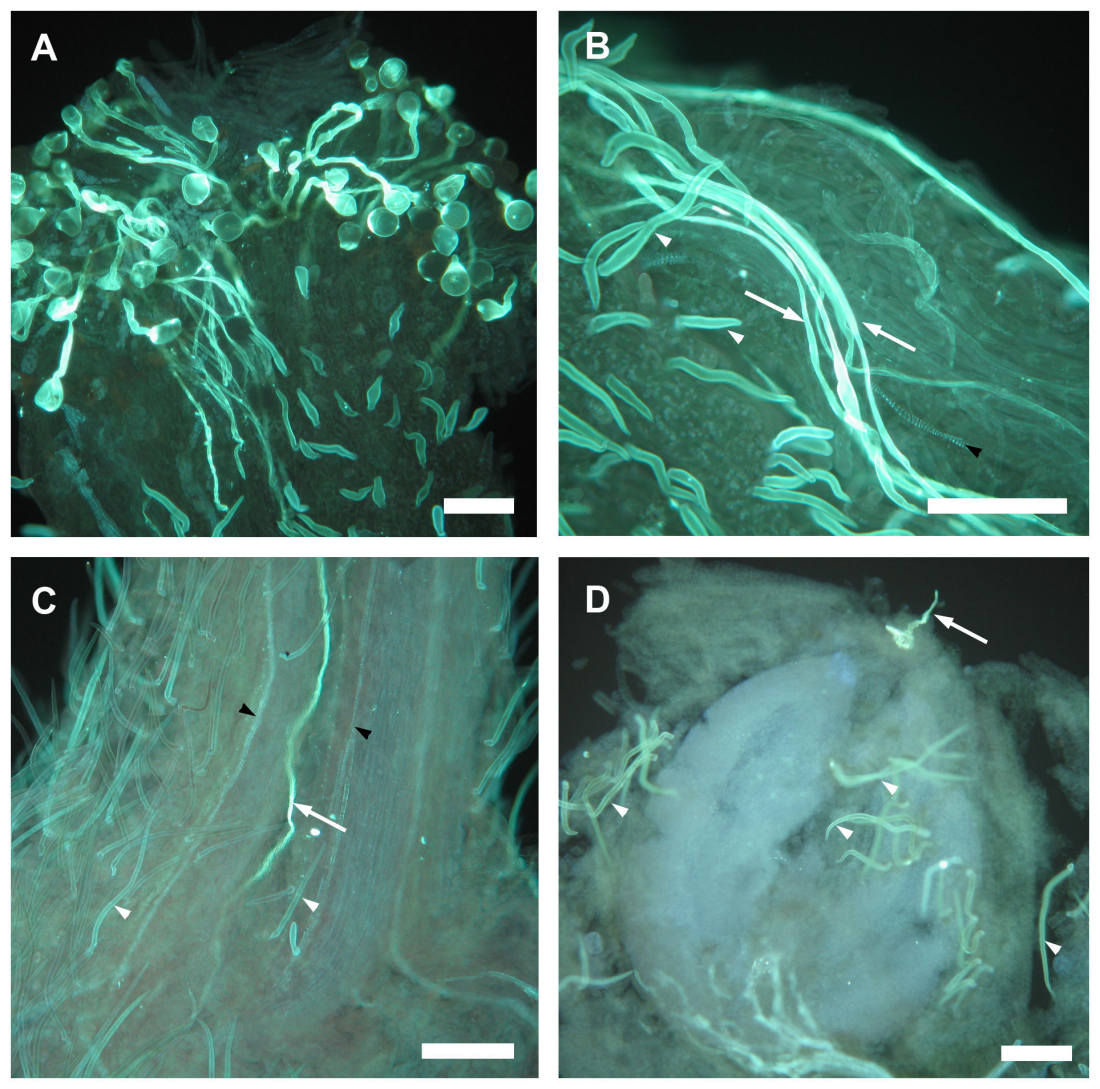
Progamic phase in avocado (*Persea americana*). Pollen germination and pollen tube growth in avocado ‘Hass’. (A) Pollen grain germination at the stigma with pollen tubes growing along the style. (B) Pollen tubes (arrows) advancing to the base of the style. (C) Pollen tube (arrow) entering the ovary. (D) Pollen tube (arrow) reaching the ovule. Other aniline blue-stained signals include trichomes (white arrowheads) and xylem vessels (black arrowheads). Scale bars = 100 μm.

 Avocado showed a rapid progamic phase in which pollen grains germinated within the first hours following pollination (Figure 2A, Table 1) and pollen tubes grew along the style within 1 day following pollination (Figure 2B, Table 1). The number of pollen tubes reaching the different regions of the style varied depending on the time after pollination (between*within effects: F_8.7,112.9_ = 2.48 ; G-C adjustment *P* = 0.014). Pollen tubes started to reach the base of the style between 4 and 6 h after hand pollination (Figure 2C, Table 1), and, in all the flowers analysed, one pollen tube was observed entering the ovary between 8 and 24 h after pollination (Figure 2D, Table 1) . Therefore, we concluded that styles could be excised without affecting pollen tube growth one day after pollination. 

**Table 1:** Number of pollen tubes at each level of the style at different hours after pollination in flowers of avocado ‘Hass’.

**Time (Hours)**	**1/4 Style**	**1/2 Style**	**3/4 Style**	**4/4 Style**
2	3.6 ± 1.0 a	2.2 ± 0.4 a	1.2 ± 0.4 a	0 a
4	3.2 ± 1.7 ab	2.5 ± 1.1 ab	1.5 ± 0.7 a	0.7 ± 0.5 b
6	4.7 ± 1.8 ab	2.6 ± 1.3 ab	1.5 ± 0.7 a	0.7 ± 0.5 bc
8	5.5 ± 2.1 b	3.8 ± 1.7 b	1.8 ± 0.8 a	1.0 ± 0 c
24	4.5 ± 1.8 ab	2.7 ± 1.0 ab	1.2 ± 0.4 a	1.0 ± 0 c
30	4.2 ± 1.4 ab	2.2 ± 0.8 a	1.5 ± 0.5 a	1.0 ± 0 c
48	3.6 ± 1.7 ab	2.1 ± 0.8 a	1.3 ± 0.5 a	1.0 ± 0 c

Means followed by different letters in the same column are significant different (*P* < 0.05) by the Tukey test.

Values shown are the means ± s.e.

### Starch content in early and late flowering inflorescences

**Figure 3:**
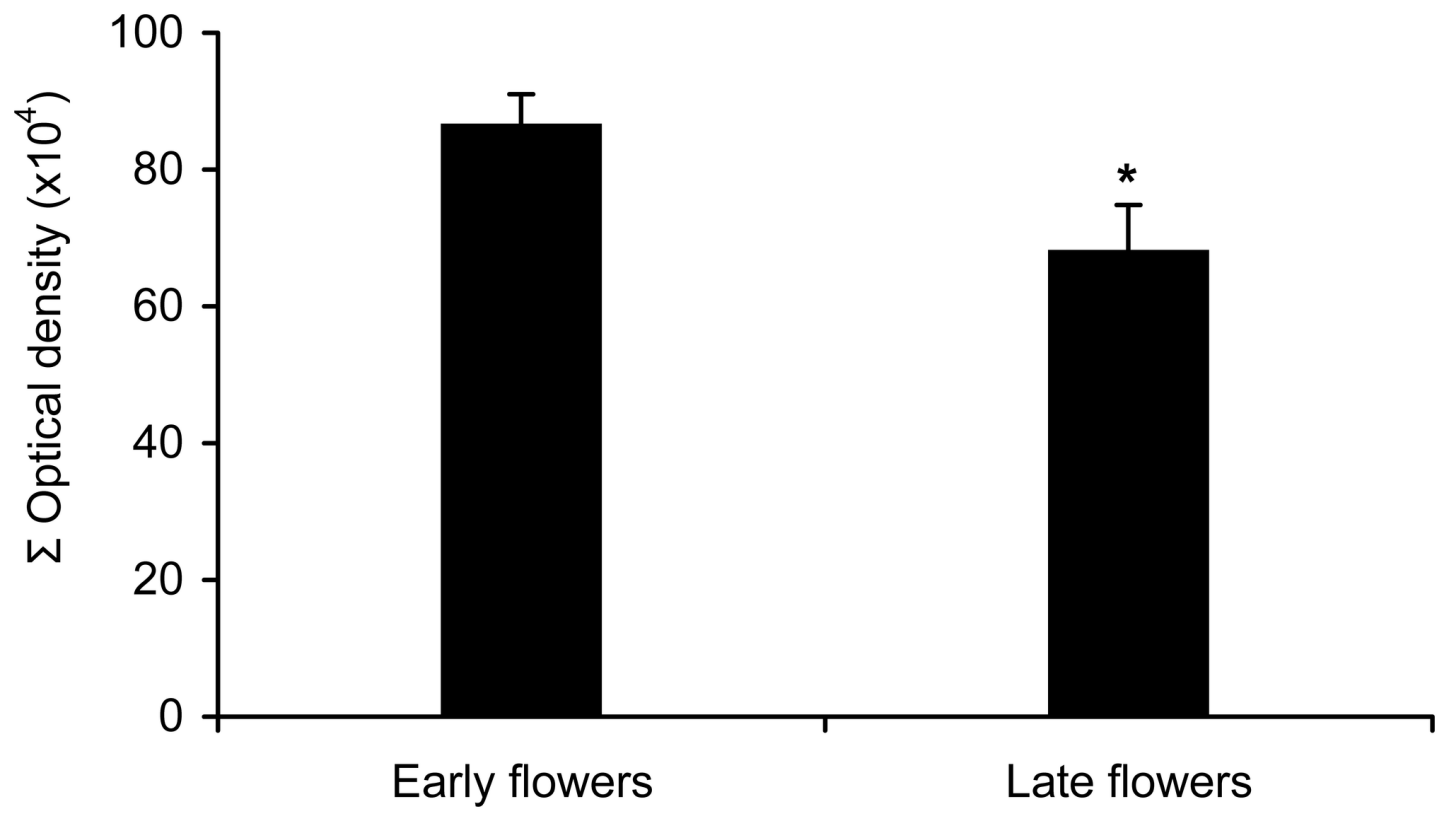
Starch content in early and late flowers. Starch content in the ovary of flowers from early-developing inflorescences with that of flowers from a group of late-developing inflorescences that open around a month later and usually do not produce fruits in avocado ‘Hass’. Mean ± SE of the average values. Asterisk indicates a significant difference (*P* < 0.05).

From a total of 10,300 open-pollinated flowers from early-developing inflorescences, 23 developed into fruits (0.22%), whereas no fruits developed in the late-developing inflorescences. The analysis of the starch content in the ovary revealed that the amount in early-developing inflorescences (867,544 Σ Optical density) was significantly higher than that in late-developing inflorescences (682,994 Σ Optical density) (*t* = 2.196, df = 87, *P* = 0.033) (Figure 3). 

### Evolution of starch content in the style after anthesis

**Figure 4:**
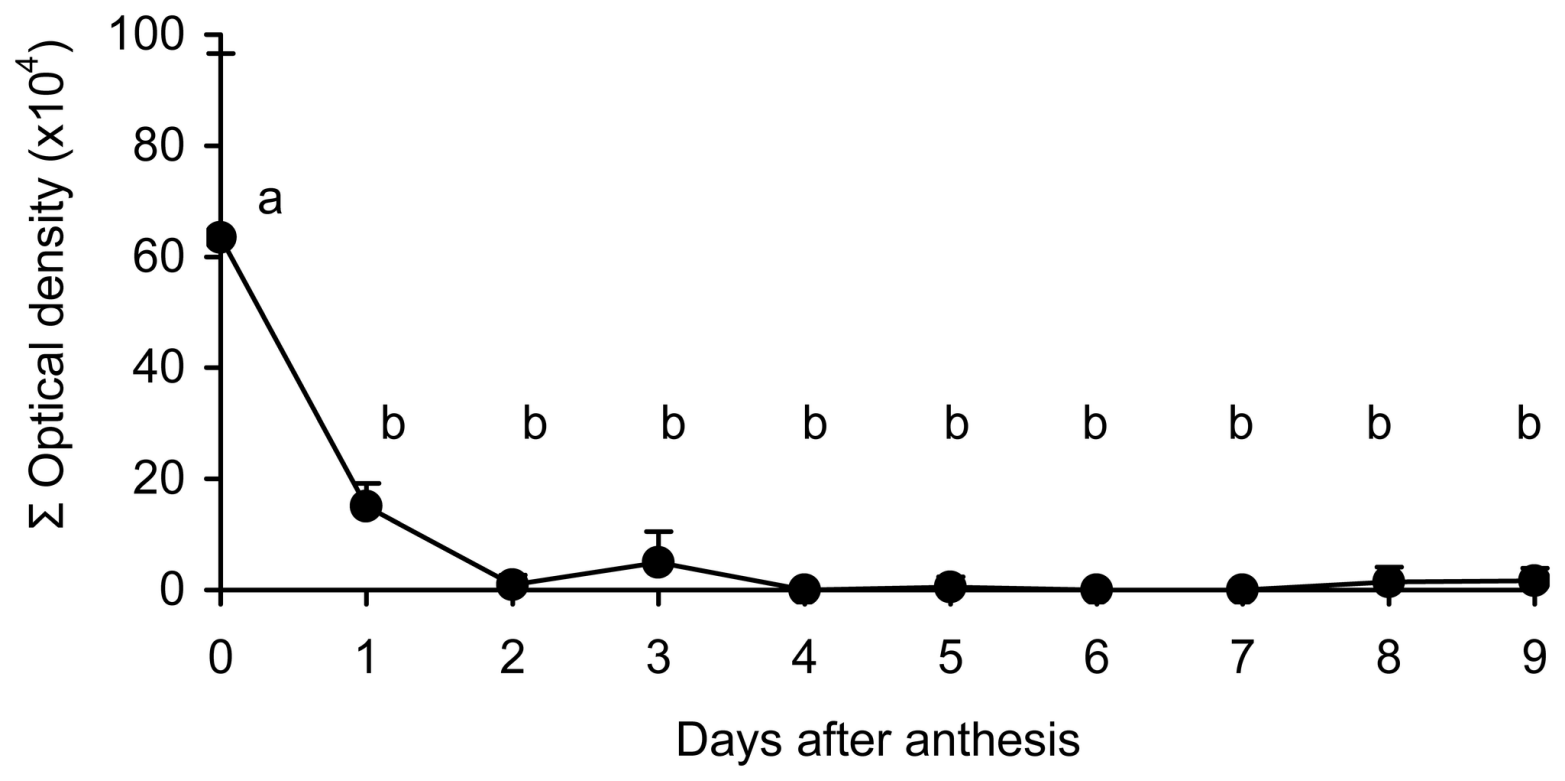
Starch content in the style of avocado flowers after anthesis. Starch content in the cortical tissue of the style in hand-pollinated flowers in avocado ‘Hass’ during 9 days following anthesis. Each value is the mean ± SE. Different letters indicate significant differences (*P* < 0.05) using the Tukey-Kramer test.

**Figure 5:**
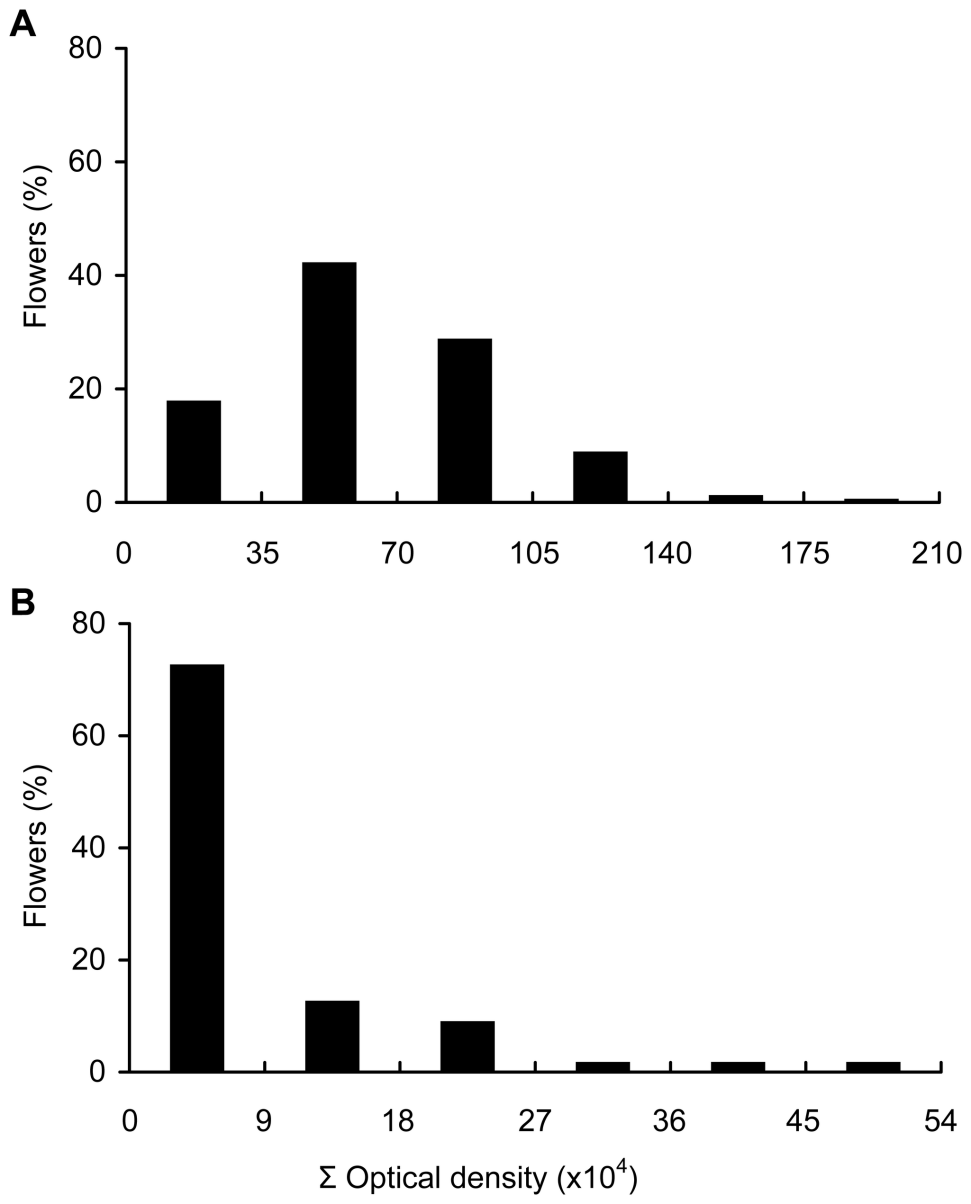
Distribution of avocado flowers according to their starch content in the style. Starch content in the style (A) at anthesis and (B) the day following anthesis in avocado ‘Hass’.

**Figure 6:**
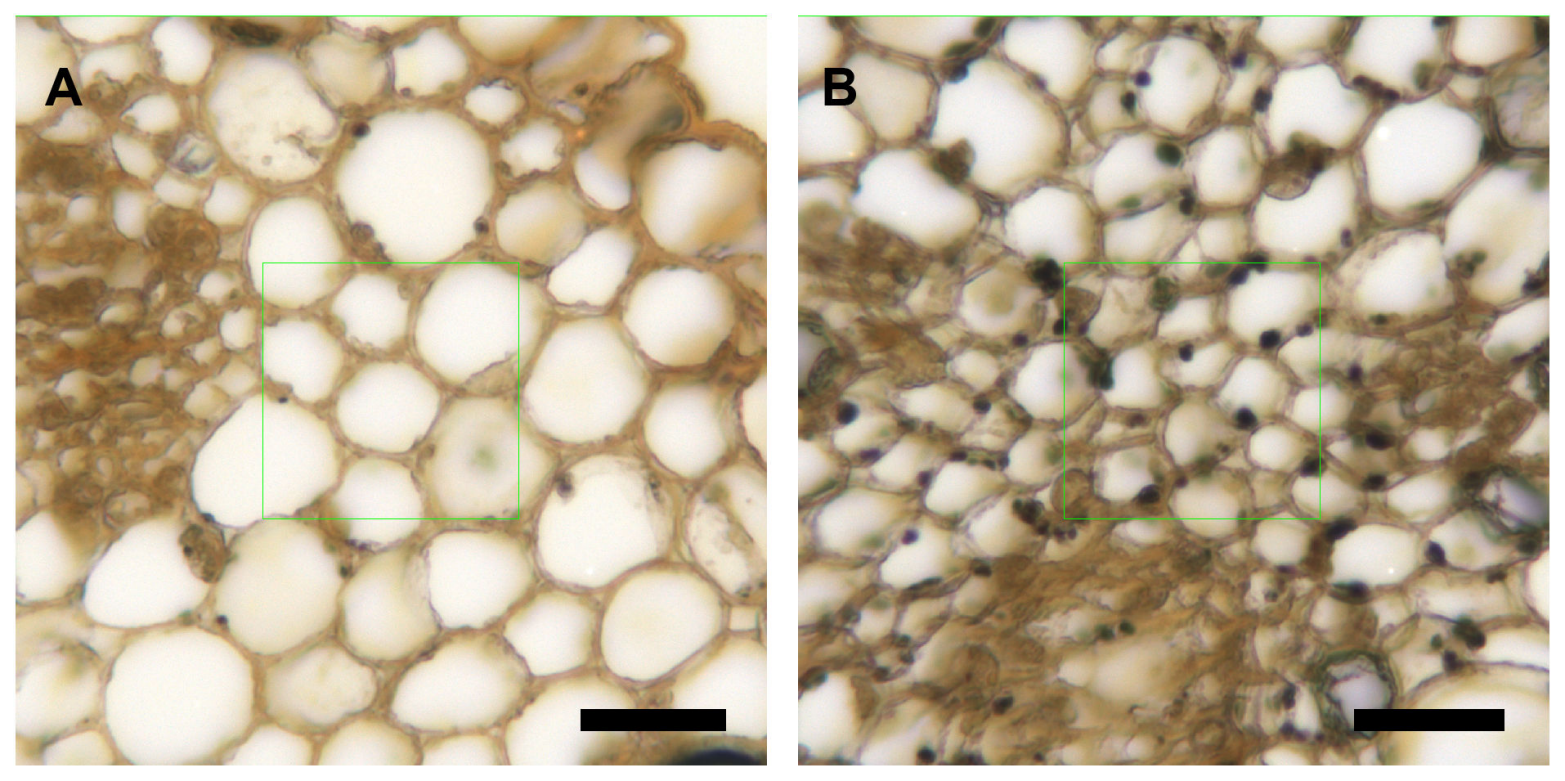
Starch in the style of avocado flowers. I_2_KI-stained sections of avocado ‘Hass’ flowers with low- (A) and high-starch content (B) in the style. Bars = 20 μm.

 At anthesis the cells of the style were rich in starch. Starch content in the style decreased in the days following anthesis (F_9,43_ = 13.04, *P* < 0.001). Such decrease was significant only one day after anthesis, while no significant changes occurred afterwards (Figure 4). However, the content of starch at anthesis was highly variable among flowers, with some flowers having starch content up to 1,000 times higher than others. Thus, the optical density values ranged from 1,866 to 1,843,127 (Figure 5A). One day after anthesis, when the style was excised from the ovary, no visible external differences were found between flowers, but high differences in the amount of starch in the style were observed under the microscope. While in some styles starch was absent (Figure 6A), starch was still present in others (Figure 6B). Starch content of the style on the day following pollination also showed a wide range of variation, ranging from a minimum value of 244 to a maximum value of 496,127 (Figure 5B).

### Correlation of starch content among the different structures of the pistil

A positive correlation between the starch content in the cortical tissue of the style and that of different parts of the pistil (cortical tissue, integuments and nucellus in both the upper and lower sections of the ovary as well as the transmitting tissue of the style) was found (Table 2). Thus, the starch content of the style was considered as a good indicator of the starch content in the pistil of each flower.

**Table 2:** Pearson correlation coefficient between the starch content in the cortical tissue of the style and different parts of the upper and lower sections of the ovary and the transmitting tissue of the style at anthesis in avocado ‘Hass’.

**Tissue**	**Pearson correlation coefficient**	**P**
**Upper section ovary**		
Cortical tissue	0.41	0.000
Integuments	0.23	0.004
Nucellus	0.19	0.007
**Lower section ovary**		
Cortical tissue	0.49	0.001
Integuments	0.23	0.004
Nucellus	0.42	0.018
**Style**		
Transmitting tissue	0.22	0.001

### Relationship between starch content in the style and fruit set

**Figure 7:**
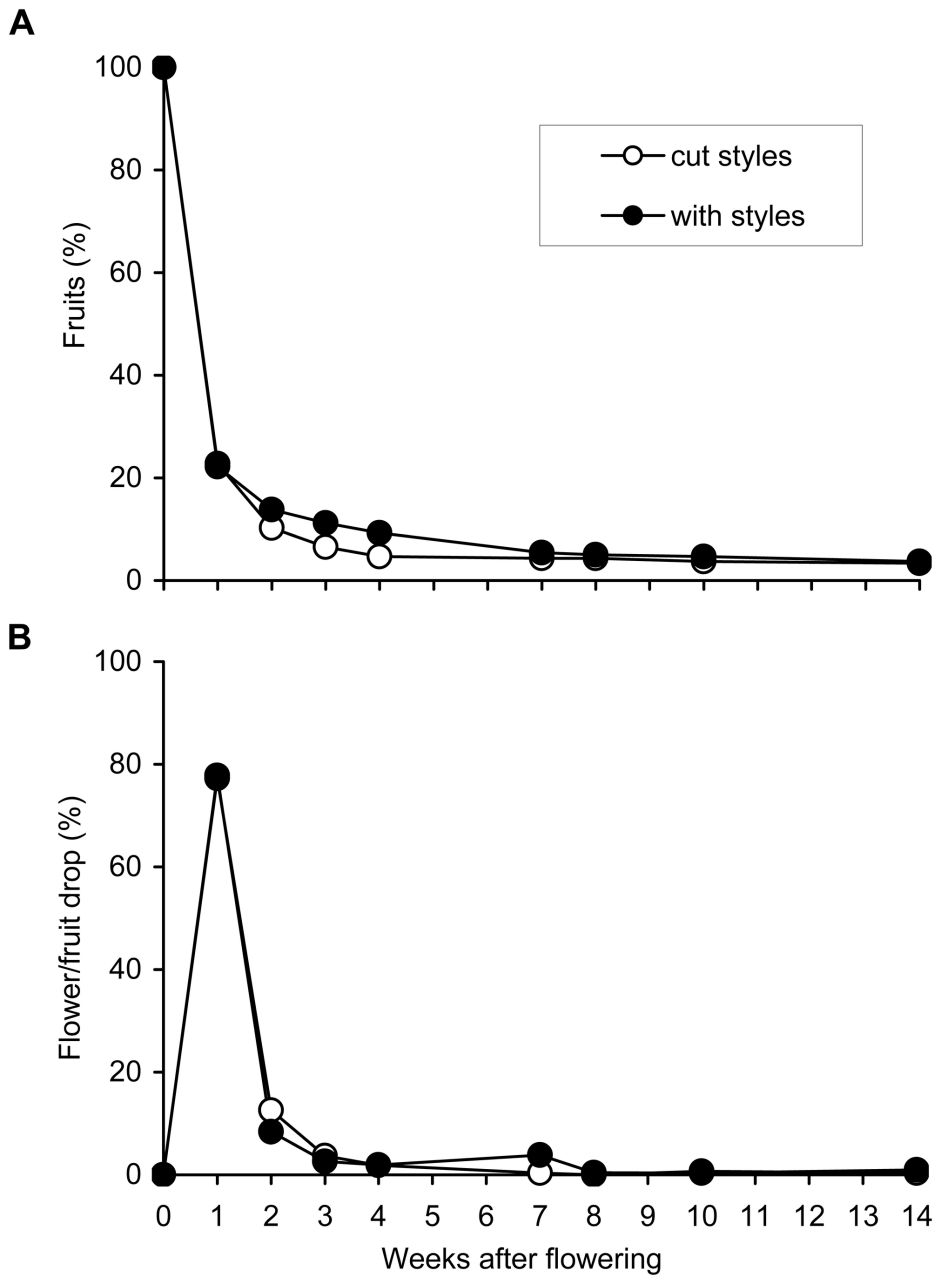
Fruit abscission in avocado. (A) Percentage of fruits remaining in the trees from the original number of flowers and (B) percentage of fruits dropped each week in relation to the initial number of flowers in a population of flowers left as control and in the population of flowers whose styles were excised 1 day after pollination in avocado ‘Hass’.

From a total of 597 hand-pollinated flowers whose styles were excised the day following anthesis, only 20 developed into fruits (3.3%). On the other hand, from the 645 hand-pollinated flowers, without dissection of the styles used as control, only 13 developed into fruits (2%). When the two populations of flowers, with or without style dissection, were compared, no differences were found either in the drop pattern (Figure 7A), or in the percentage of fruit set (*N* = 1242, *χ* ^*2*^_1_ = 1.650*, P* = 0.199). In both populations of flowers, the highest drop wave took place during the first week after anthesis (Figure 7B).

**Figure 8:**
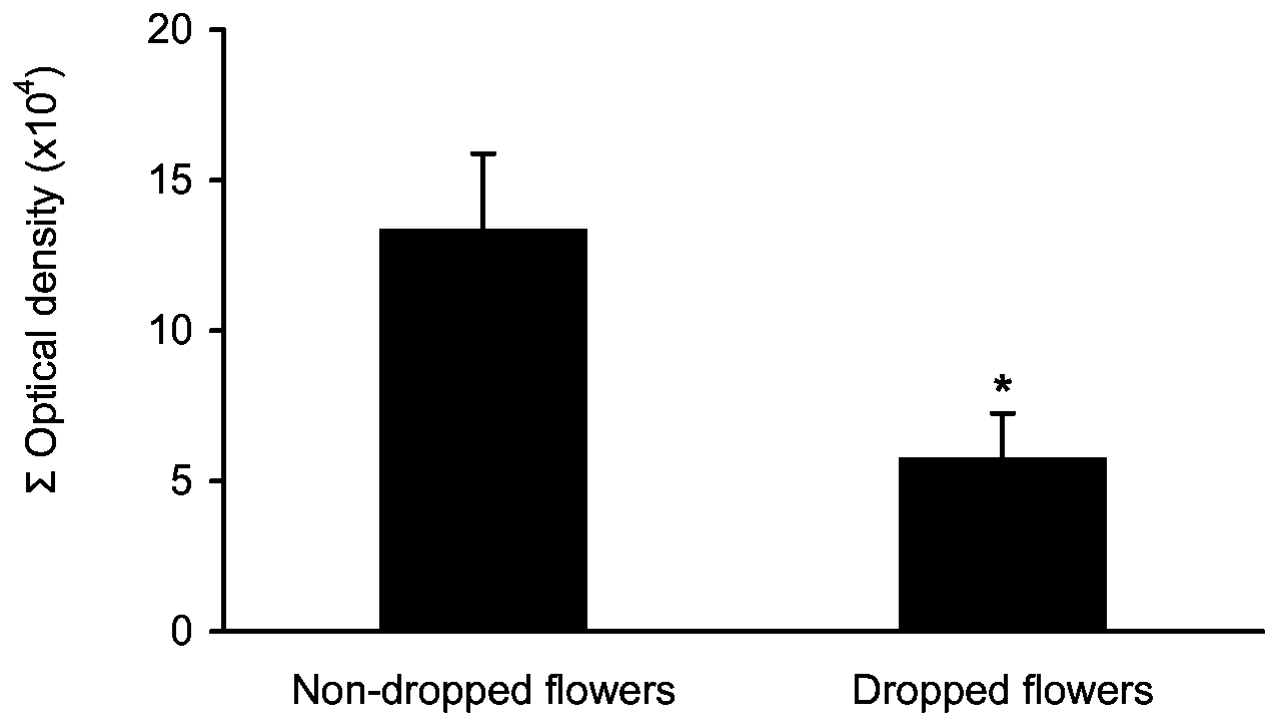
Starch content and reproductive success. Starch content in the style in two populations of flowers, those that dropped and those that remained in the trees until harvest in avocado ‘Hass’. Mean ± SE of the average values. Asterisk indicates a significant difference (*P* < 0.05).

Finally, the starch content in each excised style was related to the subsequent set or abscission of the corresponding ovary that remained on the tree. The flowers that successfully developed into fruits presented significantly higher starch content in the style (*t* = -2.64, df = 53, *P* = 0.011) than those that abscised prematurely (Figure 8). 

## Discussion

 Little is known about the ultimate factors that result in the very low percentage of fruit set in avocado even after adequate pollination. The massive abscission of flowers and developing fruits that took place from anthesis until several weeks after the end of flowering resulted in less than 1% of the fruits remaining on the trees in flowers left to open pollination [44]. The occurrence of an excess of flower production and a low fruit-to-flower ratio is a common phenomenon in hermaphrodite plants [1,48]. In this work, hand-pollination increased fruit set up to 3%, suggesting that a lack of adequate pollination could be one of the factors responsible for low yields in avocado [26]. However, even after hand-pollination, a massive drop of flowers and fruitlets still took place, suggesting that additional factors may also be involved. 

 Among the putative additional factors implicated in flower/fruit abscission, competition for carbohydrates has been shown to play an important role in different plant species [10,11,38,49]. In woody perennial plants, flower development is influenced by carbohydrate reserves stored in the tree [49] and in the pistil [17]. Vegetative growth and both flower and fruit development demand large amounts of energy. Results in citrus have shown that the reserves of carbohydrates are utilized to sustain the initial reproductive development [50], while photosynthesis from mature leaves is mainly used for vegetative growth [51]. Moreover, the role of carbohydrates on fruit set has been known for a long time. Traditional growing practices, such as girdling [52], have been used with the objective of increasing carbohydrate supply by avoiding carbohydrate mobilization to other sinks in the tree. Results in diverse fruit tree crops such as apple [53], citrus [54] or avocado [55] show a positive correlation between girdling and fruit set and it would be of interest to assess the effect of this practice on carbohydrate content in individual flowers. 

 Thus, as a first step in order to evaluate the implications of starch stored in the avocado flowers at anthesis on reproductive success, in this work differences in starch content between two populations of flowers with different reproductive success (from early and late- developing inflorescences) were studied. The results show significant differences in starch content between those two populations of flowers suggesting a role of starch, present in the flowers at anthesis, in reproductive success in avocado. Similar evidence suggesting that the flower capacity to accumulate carbohydrates is related to the subsequent fruit set or flower/fruitlet abscission is accumulating in different woody perennial plant species. Thus, starch content in the pistil at anthesis could be a good indicator of flower quality [56], an elusive term that has been used to explain differences in fruit set without an apparent ultimate cause [57]. In apricot, the stored carbohydrates in the pistil support initial fruit development [17] and appear to be related to flower fate because starch is present in the style in all the flowers that develop into fruits but only in some of the flowers that abscise [22]. In olive, hermaphrodite flowers that keep growing in the tree accumulate starch reserves, while no starch is present in the developing pistils that finally will abort before anthesis in the functionally staminate flowers [5]. In grapevine, the lack of sugar availability in flowers has been related to flower abortion [13], and differences in starch content have been reported among flowers of different genotypes with different abscission rates [20]. Likewise, high variability in starch content among flowers of the same genotypes at anthesis has been reported in apricot [17] and avocado [43]. In avocado, early fruit abscission has been attributed to low carbohydrate reserves in the trees [58] and fruit drop is accompanied with the reduction of carbohydrate export from the leaves [59]. 

However, most of the studies performed on carbohydrate metabolism and partitioning in woody perennial species have been focused on changes at the whole tree level comparing different structures [10,60] and most of the evidence is circumstantial. One of the main problems with evaluating the effect of the starch present in the flowers at anthesis on final reproductive success is that the analyses or the nutritive status of the flowers are destructive and, consequently, the analysed flowers are not available to assess their reproductive success. In order to overcome this problem in this work we used the approach of dissecting the styles once the pollen tubes have gone through. This method of style removal after pollen tube passage did not show any significant effect on flower/fruit abscission. A similar approach has previously been used to evaluate the effect of pollination intensity on fruiting in cacao [61] and to explore the implications of the stigma and style traits in the development of the ovary in apricot [22]. Additional work is under way to develop alternative analytical approaches, such as mass spectrometry, to measure starch and other compounds in detached styles, but the main limitation of these alternative techniques is the small amount of tissue available for analyses. Regarding the genes involved, in higher plants, at least 3 enzymes have been found to be directly involved in starch biosynthesis: ADP glucose pyrophosphorylases, starch synthases and starch branching enzymes. Some of them such as sucrose synthase have been shown to be active in flowers [62] and could be of interest to study the differential expression of them among different flowers. 

The results obtained in this work allowed us to establish differences among flowers in the same tree and even in the same inflorescence. Avocado flowers with a higher starch content in the pistil were found to successfully develop into fruit whereas those with a low starch content abscised prematurely. Therefore, the amount of starch in the avocado pistil appears to play an important role in fertilization and fruit set as has been suggested in other species [17,20,24], indicating that the capacity of a flower to become a fruit could be predetermined by its nutritive status at anthesis. Nevertheless, the starch content of the flower might be considered as a prerequisite to assure fertilization and posterior fruit development since, for an optimal yield, a sequence of processes including pollination, pollen grain germination, pollen tube growth, fertilization and fruit initiation must take place successfully. Understanding the process of starch accumulation in the reproductive structures from early bud differentiation until flowering could open the way to elucidate the mechanisms behind the observed differences in flower quality at anthesis not only in avocado but also in other plant species with a low fruit to flower ratio. Indeed the particular situation of avocado as one of the few crops included among the basal angiosperms provides the results obtained with significant relevance from an evolutionary point of view. The availability of genetic transformation protocols and genomic tools in this species could be highly useful to understand the ultimate developmental genetic factors behind the low fruit to flower ratios observed.
